# Single‐Cell RNAseq Identifies Heterogeneity in Myoblasts From Older Adults With Differences Related to Muscle Mass and Function

**DOI:** 10.1002/jcsm.70213

**Published:** 2026-02-17

**Authors:** Mark A. Burton, Emma S. Garratt, Hanan Y. Sharkh, Matthew O. Hewitt, Elie Antoun, Leo D. Westbury, Elaine M. Dennison, Nicholas C. Harvey, Cyrus Cooper, Harnish P. Patel, Keith M. Godfrey, Karen A. Lillycrop

**Affiliations:** ^1^ Human Development and Health Academic Unit, Faculty of Medicine University of Southampton Southampton UK; ^2^ NIHR Southampton Biomedical Research Centre University of Southampton & University Hospital Southampton NHS Foundation Trust Southampton UK; ^3^ Biological Sciences University of Southampton Southampton UK; ^4^ MRC Lifecourse Epidemiology Centre University of Southampton Southampton UK; ^5^ NIHR Oxford Biomedical Research Centre University of Oxford Oxford UK; ^6^ Academic Geriatric Medicine, Faculty of Medicine University of Southampton Southampton UK

**Keywords:** ageing, cell heterogeneity, myoblasts, sarcopenia, single‐cell transcriptomics, skeletal muscle

## Abstract

**Background:**

Ageing is associated with the loss of muscle mass and function, with consequences for metabolic health, frailty and independence in later life. The aim of this study was to investigate the transcriptional heterogeneity of human proliferating muscle satellite/stem cells (myoblasts) from older adults and how this heterogeneity may vary between healthy individuals and those with low muscle mass and function.

**Methods:**

Single‐cell transcriptomic analysis was carried out on proliferating myoblasts isolated from *vastus lateralis* biopsies from 132 participants (34 male, 98 female) aged 72–83 years from the Hertfordshire Sarcopenia Study extension. Uniform Manifold Approximation and Projection (UMAP) clustering was applied to identify clusters of myoblasts with distinct transcriptional profiles, Gene Ontology analysis was used to identify pathways enriched among the clusters, and pseudotime trajectory analysis was used to identify inferred cell lineages. Differential gene expression within cell clusters, together with the proportions of cells within each cluster and lineage, were assessed with respect to participant appendicular lean‐mass index (ALMi), grip strength, and gait speed.

**Results:**

Thirteen distinct cell clusters based on the transcriptional heterogeneity of the myoblasts were identified. Clusters 0–6 contained the majority (94.6%) of cells. Marker genes were enriched for cytoplasmic translation (Cluster 0, false discovery rate [FDR] = 7.21 × 10^−63^), muscle development (Cluster 1, FDR = 2.25 × 10^−13^), cell proliferation (Clusters 2, 4 and 6, all FDR ≤ 0.05), extracellular matrix organisation (Cluster 3, FDR = 1.92 × 10^−45^) and RNA processing (Cluster 5, FDR = 1.89 × 10^−08^). Individuals with the highest grip strength and ALMi had a greater proportion of Cluster 1 and Cluster 5 cells. Gene expression analysis (FDR ≤ 0.05) within the clusters identified 22 differentially expressed transcripts with respect to ALMi in Cluster 2 and 13 with respect to grip strength in Cluster 1. Inferred lineage analysis identified cells transitioning along five trajectories (L1–L5), including cells in L1, L3 and L4 progressing towards a stressed pre‐senescent/senescent (L1) or fibrogenic (L3 and L4) state, with cells in these lineages being more likely to originate from individuals with low ALMi (χ^2^ *p* = 1.11 × 10^−146^) and grip strength (χ^2^ *p* = 1.31 × 10^−269^).

**Conclusion:**

Our findings demonstrate considerable transcriptional heterogeneity in skeletal muscle myoblasts from older adults. This heterogeneity includes myoblasts from individuals with low muscle mass and strength progressing towards a fibrogenic or stressed state.

## 1 Introduction

Decline in muscle mass and function with ageing is associated with concomitant changes in metabolic health, frailty and independence during later life. Reduced muscle mass and strength termed sarcopenia [1] is defined by various operational definitions used worldwide [2, 3, 4], based on threshold values for lean muscle mass, grip strength and gait speed.

Mechanisms that drive decline in muscle mass and strength with ageing are considered multifactorial, with lifestyle factors, systemic and molecular perturbations implicated [5]. It is unclear how changes in molecular mechanisms lead to muscle dysregulation, resulting in variable rates of muscle decline during older age. Age‐linked muscle atrophy has been attributed, in part, to the diminishing ability of muscle to self‐repair [6], which depends on specialised stem cells (satellite cells [SCs]), located below the basal lamina of the myofibre. Upon injury, SCs activate and proliferate (termed myoblasts). Myoblasts then adopt one of two fates: differentiating into myotubes or returning to a quiescent state to repopulate the SC pool [7]. Many studies have demonstrated age‐related decline in the number and function of SCs [8, 9, 10]. However, their precise roles in the loss of muscle mass and strength during older age remain controversial [11].

It is becoming clear that muscle SCs (MUSCs) are not a homogeneous population of cells [12]. Recently, human studies have demonstrated considerable transcriptional heterogeneity of MUSCs [13]. Freshly isolated skeletal muscle *Pax7* + SCs from a range of muscle types exhibit diverse transcriptional heterogeneity and can be ordered in various states of transition, from stem‐like cells to more differentiated progenitors [14]. Furthermore, single nuclear transcriptome analysis of muscle tissue reported four populations of MUSCs [15]. MUSCs from aged versus young individuals exhibit distinct differences, with a subcluster of SCs expressing senescence markers increasing in frequency in samples from older participants [16].

The rate of muscle loss during older age varies considerably between individuals. There are limited human studies to date investigating transcriptional heterogeneity of aged SCs/myoblasts and how this may vary in individuals with low muscle mass and function compared to healthy older individuals. To determine the transcriptional heterogeneity of myoblasts isolated from older adults, we investigated the single‐cell transcriptomes of myoblasts, obtained from muscle biopsies of participants from the Hertfordshire Sarcopenia Study extension (HSSe), a cohort of well‐phenotyped older adults with and without sarcopenia born and currently residing in Hertfordshire, UK, with respect to appendicular lean‐mass index (ALMi) and grip strength or gait speed, the three primary clinical parameters used to define later life muscle function.

## 2 Methods

### 2.1 Study Design

The Hertfordshire Cohort Study (HCS) was a UK cohort study designed to investigate lifecourse influences on muscle function in community‐dwelling older adults [17]. The original HCS consisted of 2997 participants; of these, a small subset (165) were called back some years later for muscle biopsies and extensive further phenotyping (termed HSSe cohort). One hundred forty‐six of these HSSe participants had sufficient muscle tissue for isolation and culture of myoblasts (methods reported previously [18]), of which 132 passed quality control (QC) for downstream scRNASeq analysis. This represents 80% of the HSSe cohort and 4.4% of the overall HCS. Comparison of descriptive statistics for the 132 HSSe participants with all remaining 2865 HCS participants showed broadly similar characteristics (age, BMI, smoking and social class) at baseline timepoint when the HCS was formed (Table S1a–c). Individuals were actively excluded if they had diabetes, neuromuscular conditions affecting the legs, were on anticoagulants or had ischaemic heart disease. CKD was excluded in known cases. Participants gave written informed consent, with the study approved by the Hertfordshire Research Ethics Committee (07/Q0204/68).

### 2.2 Cell Collection, Drop‐Seq Processing and cDNA Library Experimental Pipeline

Myoblasts were thawed in three culture batches. Multiple approaches (PCA and UMAP data visualisation, Complementary Permutational Multivariate Analysis of Variance (PERMANOVA) [19], silhouette analysis, nearest‐neighbour mixing and batch entropy) indicated batch effects did not warrant adjustment (Table S2).

### 2.3 Pre‐Processing of Single‐Cell RNAseq (scRNAseq) Data and DGE Matrix Filtering

FASTQ files were processed through the Drop‐seq alignment pipeline [20] (Supplementary Methods). A digital gene expression (DGE) profile was generated for each sample, with gene expression of each cell per sample. The number of cell barcodes corresponding to single cells was determined, with barcodes corresponding to empty beads or noise discarded. Knee plots and density plots of the cumulative fraction of reads were generated for each DGE profile. The number of barcodes corresponding to actual cells was determined as the first inflection point after the initial peak. Low‐quality cells (library sizes and number of features five median absolute deviations (MADs) below the median, < 1000 transcripts and/or > 15% mitochondrial reads), genes not expressed in any cells and doublets (read counts 2 MADs above the medians) were excluded.

### 2.4 Cell Cycle Scoring, Dimensionality Reduction and Single‐Cell RNAseq Data Clustering

Cell cycle scoring, dimensionality reduction and scRNA‐seq data clustering was carried out using Seurat v3.2.2 (in R) (Supplementary Methods). Pseudobulk analysis of gene expression differences within the clusters with respect to grip strength, ALMi and gait speed was run using the DESeq2 package in R, and age and sex were adjusted for as covariates in the model. Inclusion of ribosomal genes, long non‐coding RNAs and pseudogenes within the final analysis allowed identification of cell state.

### 2.5 Pseudotime Trajectory Analysis (PTA)

Cellular trajectories were identified using Slingshot [21], with C2 cells (exhibiting the highest expression of proliferation markers) used as root nodes. Pseudotime values were assigned to individual cells based on their positions along inferred trajectories. Generalised additive models (GAMs) were used to identify genes with altered expression patterns along trajectories (tradeSeq).

### 2.6 Gene Ontology Enrichment (GO)

Gene Ontology (GO) analysis was carried out using genes identified by patternMarkers as associated with each of the learned patterns. Genes were selected based on their patternMarker statistic and used the classic Fisher test, as implemented in the topGO R package for enrichment of biological process (BP) GO terms. The 3000 most variable genes used in the latent factor modelling were used as the background gene set.

### 2.7 Statistical Analysis

Statistical analyses were performed in R v3.6.2. Appropriate standard summary statistics were applied.

## 3 Results

### 3.1 Cohort Characteristics

Table 1 shows participant characteristics for the 34 male and 98 female HSSe participants with myoblast scRNA‐seq data that passed QC. Median (IQR) age was 78.00 (3.80), appendicular lean mass (ALM) was 15.75 (4.49) kg, appendicular lean mass index (ALMi) was 5.99 (1.25) kg/m^2^, and grip strength was 23.00 (11.00) kg. Mean (±SD) gait speed was 0.97 (0.21) m/s.

**TABLE 1:** Participant characteristics.

Characteristics	Value (mean)	±SD	Median (IQR)
Number	132	—	—
Female	98	—	—
Male	34	—	—
Age (years)	78.00	—	78.00 (3.80)
Height (cm)	162.07	8.02	—
Weight (kg)	71.64	12.77	—
BMI	26.90	4.19	—
Fat mass (kg)	29.15	8.82	—
Body fat (%)	40.88	—	42.59 (9.04)
Total lean body mass (kg)	39.10	—	37.74 (8.99)
ALM (kg)	16.46	—	15.75 (4.49)
ALMi (kg/m^2^)	6.17	—	5.99 (1.25)
Grip strength (kg)	24.74	—	23.00 (11.00)
Gait speed (m/s)	0.97	0.21	—

*Note:* Values are mean ± standard deviation (SD) and median (interquartile range [IQR]) as appropriate.

### 3.2 scRNAseq of Actively Proliferating Myoblasts Identified Cellular Heterogeneity in Gene Expression

**FIGURE 1:**
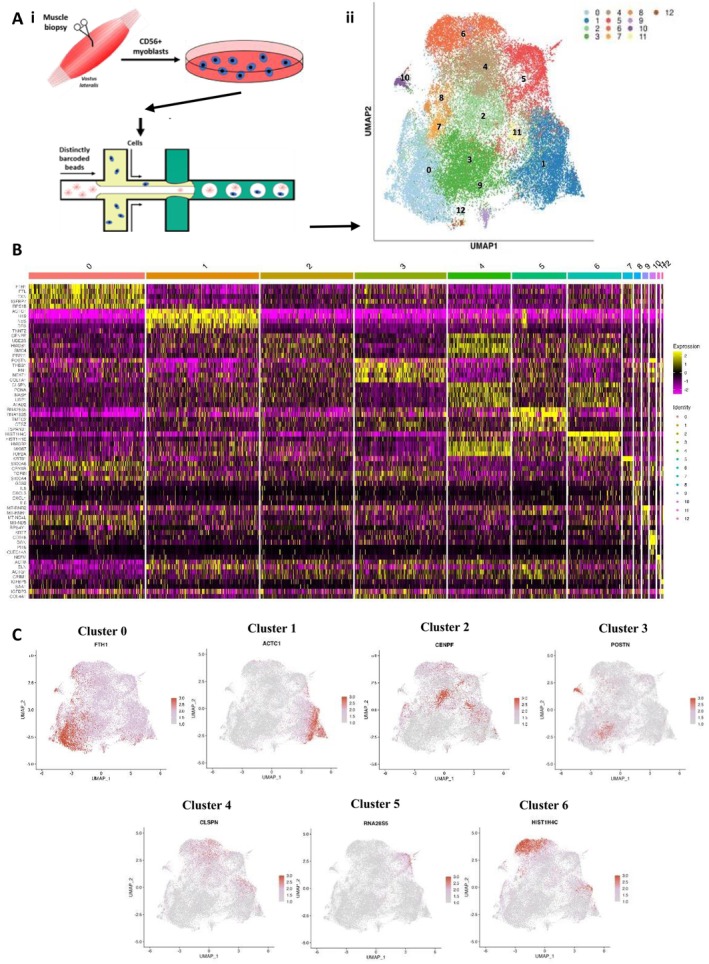
(A) (i) Schematic of experimental workflow. (ii) Uniform Manifold Approximation and Projection (UMAP) analyses identified 13 subpopulations of cells (Clusters 0–12) based on their gene expression profiles. (B) Heat map of the 13 cell clusters showing expression of the top 5 marker genes in each cluster (left) and expression of those genes combined in each cluster (C). Dot maps of the top marker genes in Clusters 0–6.

A median of 278 cells was sequenced per participant (38 635 total cells), with 2703 (median) features identified per cell (Figure 1A). UMAP identified 13 cell subpopulations based on their transcriptional expression profiles (Figure 1Aii,1B); Clusters 0–6 contained 94.6% of total cells (7280–3328 cells per cluster), with Clusters 7–12 containing fewer cells (5.4% of total cells, 632–125 cells per cluster) (Table S3). Each cluster had a unique transcriptomic fingerprint with heterogeneous gene expression (Figure 1B,C).

### 3.3 Differential Gene Expression Identified Cells at Different Stages of Myogenic Lineage Commitment

Differential expression of marker genes between cell clusters identified genes more highly expressed in particular clusters (FDR ≤ 0.05) (Table 2, Table S4a and Figure 1Aii,B).

**TABLE 2:** Top 3 marker genes for the main cell clusters 0–6.

Cluster	Gene ID	Gene name
0	FTH1	Ferritin heavy chain
0	FTL	Ferritin light chain
0	TXN	Thioredoxin
1	ACTC1	Cardiac alpha actin
1	H19	H19
1	NES	Nestin
2	CENPF	Centromere protein F
2	CENPE	Centromere protein E
2	UBE2S	Ubiquitin‐conjugating enzyme E2 S
3	POSTN	Periostin
3	THBS1	Thrombospondin 1
3	FN1	Fibronectin
4	CLSPN	Claspin
4	PCNA	Proliferating cell nuclear antigen
4	NASP	Nuclear autoantigenic sperm protein
5	RNA28S5	28S ribosomal 5
5	RNA18S5	18s ribosomal 5
5	TMTC2	Transmembrane O‐mannosyltransferase targeting cadherins 2
6	HIST1H4C	Histone cluster 1 H4 family member C
6	HIST1H1E	Histone cluster 1 H1 family member E
6	HMGB2	High mobility group box 2

#### 3.3.1 Cluster 0 (C0)

**FIGURE 2:**
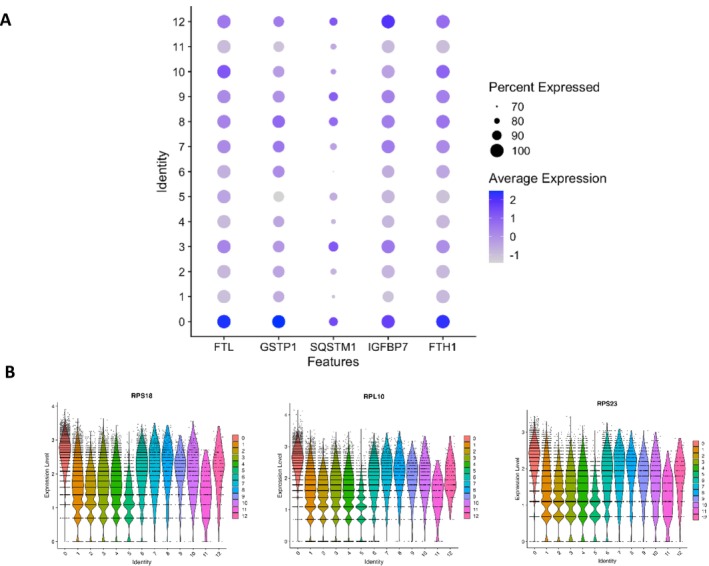
(A) Dot plot showing oxidative stress/senescence genes upregulated in Cluster 0. (B) Violin plots showing differential expression analysis of *RPS18*, *RPL10* and *RPS23* across all clusters (C0–C12) with highest expression levels in Cluster 0.

C0 (18.8% of total) cells exhibited increased expression of ribosomal genes including ribosomal protein L17 (*RPL17*) and 31 (*RPL31*), oxidative stress response genes including glutathione S‐transferase P (*GSTP1*), thioredoxin (*TXN*) and NAD(P)H quinone dehydrogenase 1 (*NQO1*) and genes involved in oxidative phosphorylation (OXPHOS) including cytochrome C oxidase subunit‐5B (*COX5B*), 8A (*COX8A*) and 6A1 (*COX6A1*) and NADH/ubiquinone oxidoreductase subunit‐B3 (*NDUFB3*) and subunit‐B6 (*NDUFB6*). Cells in C0 also expressed high levels of autophagy related genes including sequestosome 1 (*SQSTM1*) and GABA Type A receptor associated protein like 2 (*GABARAPL2*), together with senescence markers including cyclin‐dependent kinase 1A (*CDKN1A*) and insulin growth factor binding protein 7 (*IGFBP7*) (Figure 2). Further subclustering of C0 identified five subclusters (C00–04) with distinct marker gene expression profiles (Table S4b). Pathway enrichment of marker genes identified key pathways linked to oxidative phosphorylation and cellular response to stress (C00); RNA splicing and transcription coregulator activity (C01); axon guidance and cellular response to chemical stress (C02); and cell cycle mitotic and DNA metabolic process (C03). No pathways were identified for C04 (Tables S4c–S4f). The top BP pathway associated with C0 was cytoplasmic translation (FDR = 7.21 × 10^−63^) and top molecular function (MF) pathway structural constituent of ribosome (FDR = 8.82 × 10^−63^) (Table S5).

#### 3.3.2 Cluster 1 (C1)

**FIGURE 3:**
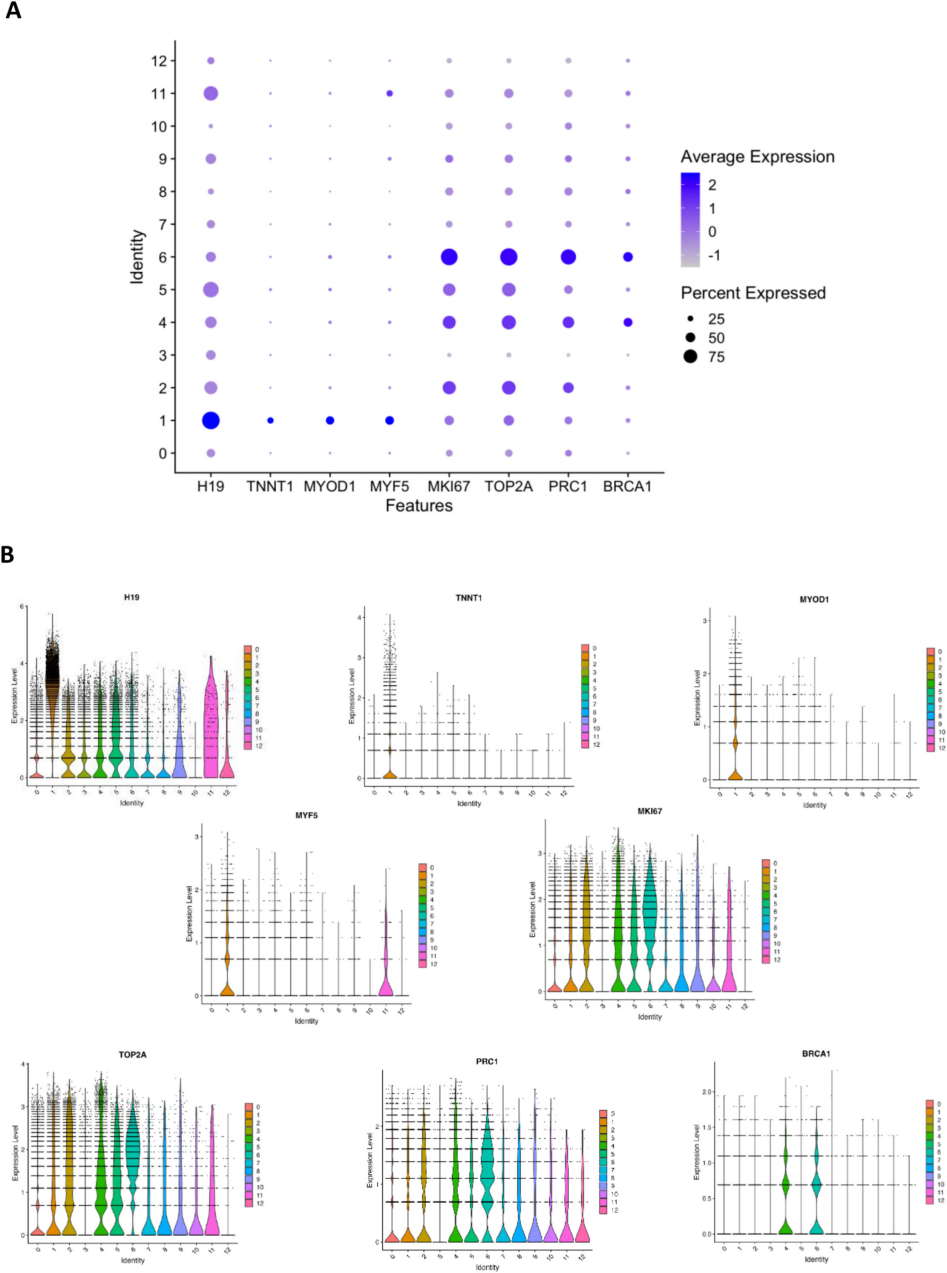
(A) Dot plot showing increased expression of cluster 1 myogenic genes (*H19*, *TNNT1*, *MYOD*, *MYF5* and *MK167*) and expression of cell proliferation markers (*TOP2A* and *PRC1*) in Clusters 2, 4 and 6. Clusters 4 and 6 expressed high levels of DNA repair genes including *BRCA1*. (B) Violin plots showing differential expression analysis of the above genes across all clusters (C0–C12).

C1 (18.3% of total) cells showed high expression of myogenic genes, including *H19*, troponin T1 slow skeletal type (*TNNT1*), myogenic differentiation 1 (*MYOD1*) and myogenic factor 5 (*MYF5*) (Figure 3). Cells also expressed the highest levels of *PAX7* compared to other clusters. For C1, marker genes were enriched for muscle tissue development (FDR = 2.25 × 10^−13^) (BP pathway) and actin binding (FDR = 1.04 × 10^−4^) (MF pathway). Similar pathways were enriched in Cluster 11 (C11) (0.45% of cells), with the top BP pathway being striated muscle cell differentiation (FDR = 8.55 × 10^−09^) (Table S5).

#### 3.3.3 Clusters 2, 4 and 6 (C2, C4, C6)

Cells in C2, C4 and C6 (15.0%, 10.2% and 8.6% of total cells, respectively) showed increased expression of cell proliferation markers, including high mobility group box 2 (*HMGB2*), marker of proliferation Ki‐67 (*MKI67*) and DNA topoisomerase II Alpha (*TOP2A*); cells in C4 and C6 also expressed high levels of DNA repair genes including breast cancer type 1 and type II susceptibility genes (*BRCA1*/*BRCA2*) and poly[ADP‐ribose] polymerase 1 (*PARP1*) (Figure 3). The top BP pathways were nuclear division (FDR = 1.12 × 10^−14^) in C2, DNA replication (FDR = 2.67 × 10^−49^) in C4 and chromosome segregation (FDR = 5.32 × 10^−57^) in C6; the top MF pathways were tubulin binding (FDR = 2.14 × 10^−08^) in C2, catalytic activity acting on DNA (FDR = 1.21 × 10^−17^) in C4 and single‐strand DNA binding (FDR = 2.13 × 10^−20^) in C6 (Table S5).

#### 3.3.4 Cluster 3 (C3)

**FIGURE 4:**
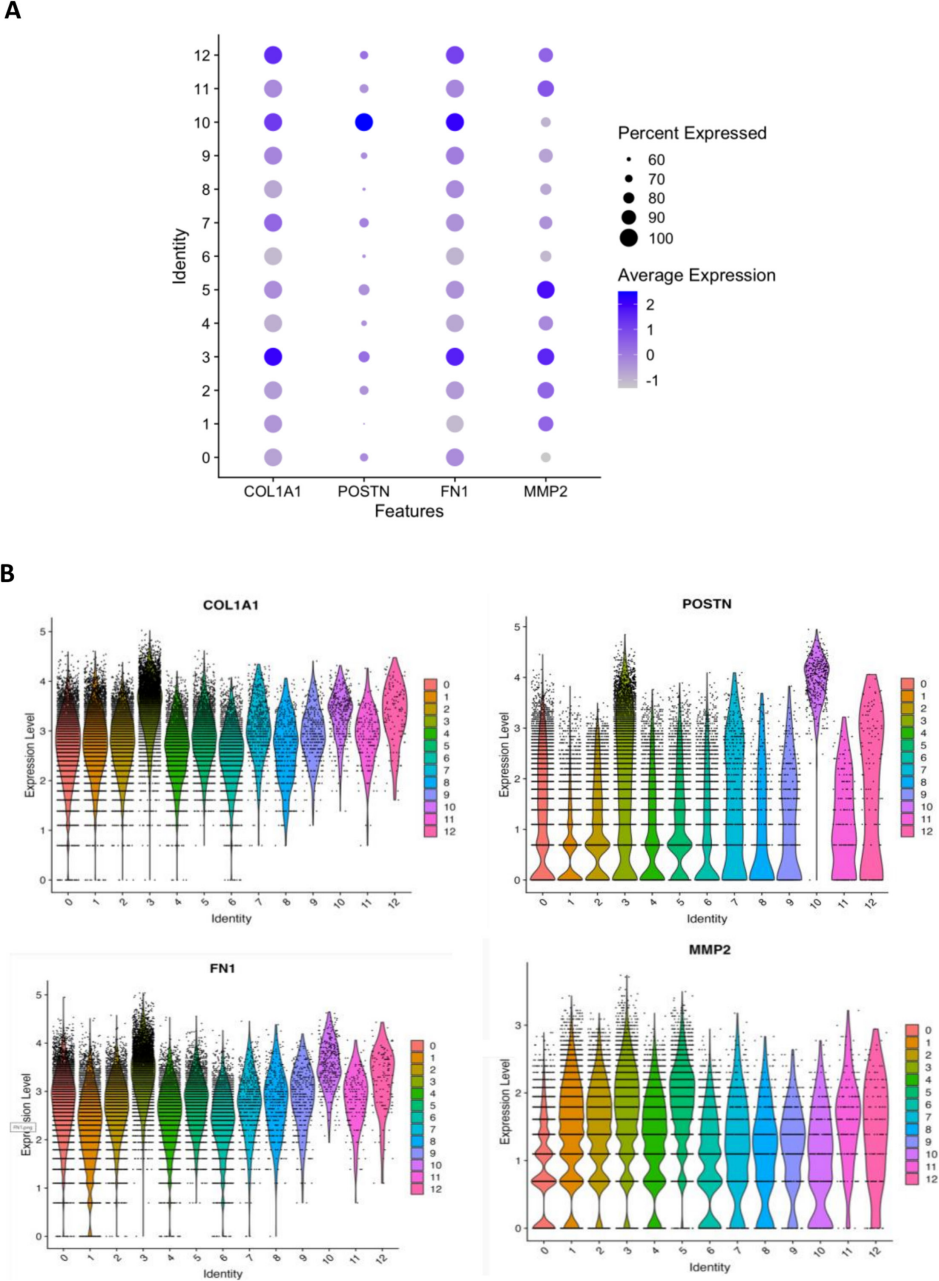
(A) Dot plot of highly expressed genes in Cluster 3. (B) Violin plots of highly expressed genes in Cluster 3 across all clusters (C0–C12).

C3 (14.9% of total) cells expressed high levels of transforming growth factor beta (TGFB1), WNT family member 5A (WNT5A) and fibroblast growth factor 2 (FGF2), alongside fibrogenic markers including periostin (POSTN), fibronectin (FN1), collagen type 1 alpha 1 (COL1A1) and matrix metalloproteinase‐2 (MMP2) (Figure 4). The top BP pathway was extracellular matrix (FDR = 1.92 × 10−45), and the top MF pathway was extracellular matrix structural constituent (FDR = 4.16 × 10−27) (Table S5).

#### 3.3.5 Cluster 5 (C5)

C5 (8.8% of total) cells had high expression of ECM components, β‐catenin (*CTNNB1*), actinin alpha‐1 (*ACTN1*) and dystonin (*DST*), together with transcriptional regulators and modifiers such as DEAD‐box helicase‐5 (*DDX5*), cathepsin Z (*CTSZ*) and polyadenylate‐binding protein 2 (*PABPN1*). The top BP pathway was mRNA Processing (FDR = 1.89 × 10^−08^), and top MF pathway was cadherin binding (FDR = 1.41 × 10^−10^) (Table S5).

#### 3.3.6 Minority Cell Clusters

C7 (1.64% of total) cells expressed ribosomal genes *RPL30* and *RPS23*, together with ECM components including *POSTN*, with the top BP pathway being cytoplasmic translation (FDR = 8.85 × 10^−04^); C8 (1% of total) cells expressed high levels of inflammatory genes including interleukin‐6 (*Il‐6*), interleukin‐8 (*Il‐8*) and nuclear factor kappa light chain enhancer of activated B cells (*NFkB2*) (Figure S1); the top BP pathway was structural constituent of ribosome (FDR = 1.53 × 10^−05^). C9 (0.97% of total) cells expressed cell morphogenesis markers including *WNT5a* and bone morphogenetic protein receptor type II (*BMPR3*), with marker genes enriched for wound healing (FDR = 8.48 × 10^−05^). C10 (0.97% of total) cells showed increased expression of keratin 7 (*KRT7*), *CD248*, biglycan (*BGN*) and peptidase inhibitor 16 (*PI16*), genes highly expressed in fibroblasts (Figure S2); cells in C10 came predominately from four participants (66% of cells originated from one individual). C12 (0.32% of total) cells expressed high levels of serum amyloid A transcript (*SAA‐1*), collagen alpha‐1(IV) chain (*COL4A1*), thrombospondin 1 (*THBS1*), secreted protein acidic and cysteine‐rich (*SPARC*), transforming growth factor beta (*TGB1*) and integrin beta‐1 (*ITGB1*), with BP pathways enriched for extracellular matrix organisation and structure (FDR = 2.69 × 10^−13^) (Table S4a).

### 3.4 Of the Major Cell Clusters, C5 and C1 Contained Cells From Individuals With the Highest Grip Strength or ALMi

**FIGURE 5:**
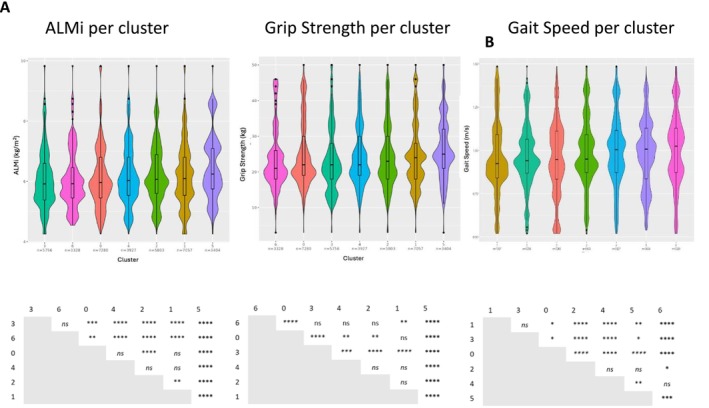
(A) Violin plots showing the distribution of ALMi, grip strength or gait speed across main cell clusters 0–6. Clusters are ordered in increasing ALMi, grip strength and gait speed. (B) Anova adjusted *p*‐values (adj p) showing differences in ALMi, grip strength or gait speed between main cell clusters 0–6 adj *p* < 0.05*, adj *p* < 0.01**, adj *p* < 0.001***, adj *p* < 0.0001****. *ns* = not significant.

To investigate the relationship between cellular distribution of clusters and muscle mass/strength/function of individuals from which they were isolated, median grip strength, ALMi and gait speed were compared for each of the major cell clusters. Cells in C5 and C1 were from individuals with the highest median grip strength, whereas cells in C6 and C0 were from the lowest grip strength. For ALMi, cells in C5 and C1 were from individuals with the highest ALMi, and C3 and C6 were from the lowest ALMi, whereas cells populating C6 and C5 had the higher median gait speed and C3 and C0 the lowest gait speed (Figure 5 and Table S6).

### 3.5 Muscle Mass, Strength and Function Were Associated With Differential Gene Expression Within Cell Clusters

To gain further insights into how differences in muscle mass and function may affect transcriptional profiles of myoblasts within different clusters, we examined gene expression differences between clusters in individuals from the highest quartile compared to the lowest quartile of grip strength, ALMi and gait speed. Pseudobulk analysis of gene expression differences within clusters revealed significant changes in levels of expression (Table S7a). For grip strength, 13 genes were differentially expressed (FDR ≤ 0.05) in C1 including aurora kinase B (AURKB, FDR = 3.66 × 10−03). For ALMi, differentially expressed (FDR ≤ 0.05) genes included three in C0, including target of Myb protein‐1 (TOM1 FDR = 3.88 × 10−02); 22 in C2, including poly(A) binding protein nuclear‐1 (PABPN1, FDR = 1.24 × 10−02); 4 in C4, including ubiquitin‐conjugating enzyme E2S (UBE2S, FDR = 2.00 × 10−02); and 2 in C5 (phosphorylase kinase regulatory subunit beta) (PHKB, FDR = 7.40 × 10−07) and casein kinase 1 gamma 1 (CSNK1G1, FDR = 4.78 × 10−08). For gait speed, four genes were differentially expressed in C5 including CSNK1G1 (FDR = 8.22 × 10−03) (Table S7a). Pathway analysis on the 13 differentially expressed genes significantly correlated with grip strength identified enrichment in cell cycle‐mitotic, positive regulation of cytoskeleton organisation and chromatin remodelling pathways (Table S7b).

### 3.6 Actively Proliferating Myoblasts Existed Along Five Different Pseudotime Lineages

**FIGURE 6:**
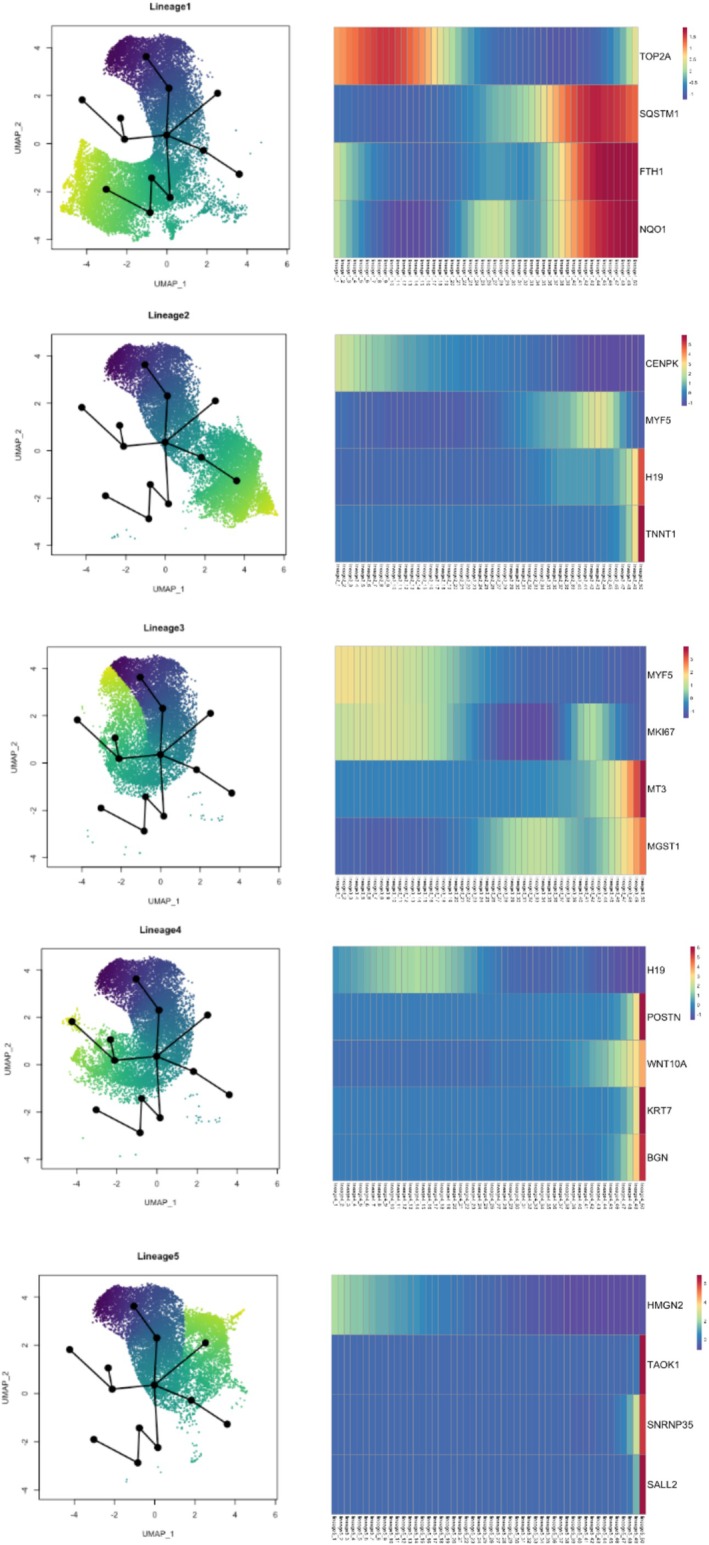
Myoblast cell lineages identified using pseudotime trajectory analysis. Cells in Cluster 6 were used as the root node from which to begin pseudotime analysis as these cells exhibited the highest average expression of early cell proliferation markers relative to the other cell clusters. From this root node, all cells progress through Cluster 4 and into Cluster 2, and the cells then diverge into five different lineages (L1–5). Corresponding heat maps to each lineage showing associated lineage specific genes and their subsequent direction and level of expression.

Differences in marker genes between clusters suggested cells were at different stages of commitment along the myogenic differentiation lineage. To confirm this, we carried out pseudotime trajectory analysis (PTA). Cells in C6 were used as the root node from which to begin, as these cells exhibited the highest average expression of early proliferation markers relative to the other clusters. From this root node, all cells progressed through C4 and into C2; cells then diverged into five different lineages (Figure 6). L1 cells progressed from C2 through C3 ending in C0, whereas in L2, cells progressed from the common node in C2 into C1. Cells in L3 and L4 progressed from C2 along the same trajectory until the next node where they diverged, with cells in L3 proceeding towards C7 and C8 and cells in L4 progressing towards C10. L5 cells progressed from the common node in C2 into C5 (Figure 6).

To investigate transcriptional differences, as cells progressed along lineages, we compared gene expression profiles at the start and end of each lineage (Table S8). Cells in L1 revealed a decrease in cell proliferation markers *TOP2A* (FDR = 8.96 × 10^−03^) and *HMGN2* (FDR = 1.06 × 10^−04^), whereas autophagy genes such as *SQSTM1* (FDR = 7.45 × 10^−27^), genes involved in regulating oxidative stress such as *FTH1* (FDR = 5.88 × 10^−23^), *NQO1* (FDR = 4.67 × 10^−10^) and heat shock protein *DNAJB4* (FDR = 1.82 × 10^−21^) were upregulated (Figure 6). In L2, we observed a downregulation of cell proliferation markers *HMGN2* (FDR = 8.68 × 10^−47^) and *CENPK* (FDR = 2.98 × 10^−09^) and a significant upregulation in expression of myogenic differentiation markers including *TNNT1* (FDR = 5.26 × 10^−53^), *TNNT2* (FDR = 1.14 × 10^−64^), *NCAM1* (FDR = 1.71 × 10^−75^), *H19* (FDR = 3.25 × 10^−64^) and *MyoD1* (FDR = 2.71 × 10^−33^), suggesting these cells were undergoing myogenic commitment and differentiation (Figure 6). Progression along L3 was also associated with downregulation of cell proliferation markers including *MK167* (FDR = 1.23 × 10^−05^) and *TOP2A* (FDR = 2.47 × 10^−03^), alongside early myogenic genes *H19* (FDR = 3.08 × 10^−03^) and *Myf5* (FDR = 8.73 × 10^−03^). This was accompanied by a significant increase in expression of metallothionein 3 (*MT3*, FDR ≤ 1.00 × 10^−20^), potassium channel subfamily K member 17 (*KCNK17*, FDR ≤ 1.00 × 10^−20^), EGF containing fibulin ECM Protein (*EFEMP1*, FDR = 4.69 × 10^−10^) and *MGST1* (FDR = 1.78 × 10^−05^). L4 exhibited a significant increase in expression of *POSTN* (FDR = 2.92 × 10^−28^), *WNT10A* (FDR = 2.92 × 10^−28^), *KRT7* (FDR = 1.68 × 10^−24^) and *BGN* (FDR = 2.35 × 10^−11^) consistent with cells progressing towards C10 and a more fibroblastic phenotype. Comparison of gene expression at the start and end of L5 revealed an upregulation in expression of thousand and one amino acid kinase 1 (*TAOK1*, *F*DR ≤ 0.0001), *CSNKIg1* (FDR ≤ 0.0001), small nuclear ribonucleoprotein U11/U12 subunit 35 (*SNRNP35*, FDR = 6.28 × 10^−40^) and spalt like transcription factor 2 (*SALL2*, FDR = 3.3810^−07^) and downregulation in cell proliferation marker *HMGN2* (FDR = 1.25 × 10^−24^) (Figure 6 and Table S8).

### 3.7 The Proportions of Cells in L4 and L5 Differed With Respect to Muscle Mass and Strength

To determine differences in cell proportions within each lineage with respect to muscle mass and function, cell proportions with regard to four quartiles of ALMi, grip strength and gait speed were compared. There was a difference in proportion of cells with respect to quartiles of ALMi (χ^2^ *p* = 1.11 × 10^−146^), grip strength (χ^2^ *p* = 1.31 × 10^−269^) and gait speed (χ^2^ *p* = 1.75 × 10^−111^); cells from individuals with the lowest ALMi were more likely to be in L1 (χ^2^ Pearson's residuals = 6.33) and L4 (10.51) and less likely to be in L2 (−4.43) or L5 (−11.05), whereas cells from individuals with the lowest grip strength were more likely to be in L4 (8.01), L3 (5.66) or L1 (3.015) and less likely to be in L5 (−11.32). For gait speed, cells from individuals with the lowest gait speed were more likely to be in L1 (3.99) and less likely to be in L2 (−4.56) (Figure S3 and Table S9).

## 4 Discussion

In this study, we carried out scRNAseq on human primary myoblasts from 132 adults aged 72–83 years. Unsupervised clustering identified 13 cell clusters with distinct transcriptional profiles; cells in C1 expressing early and late markers of myogenic differentiation, with a transcriptionally distinct myoblast population expressing high levels of β‐catenin, associated with individuals with the highest grip strength and ALMi. PTA identified cells transitioning along five trajectories, starting with cells expressing proliferation markers and progressing along either a committed, myogenic differentiation pathway or towards a stressed or fibrogenic state in individuals with low grip strength or low ALMi.

Of the 13 cell clusters, C0–6 contained the most cells. C0 exhibited a complex mixed phenotype with high expression of both OXPHOS and oxidative stress response genes. This may reflect a response mechanism to ameliorate effects of increased OXPHOS and production of reactive oxygen species (ROS) formation, which can induce oxidative damage to nuclear and mitochondrial DNA and induce cellular senescence. C0 cells also expressed high levels of ribosomal transcripts including ribosomal protein S7 (*RPS7*), S3 (*RPS3*) and S14 (*RPS14*), which may reflect metabolic activity and preparation for translation. However, cellular senescence is also reported to lead to diminished ribosome biogenesis and accumulation of rRNA precursors and ribosomal proteins, with overexpression of *RPS14* sufficient to inhibit cyclin dependent kinases, induce cell cycle arrest and cellular senescence [22]. Cells in C0 expressed high levels of *CDKN1A* consistent with a pre‐senescence/senescence‐like phenotype. The combination of high levels of oxidative response genes and accumulation of ribosomal transcripts suggests a heightened stressed state with oxidative damage and/or pre‐senescence changes and activation of stress‐mediated ribosomal transcription. Ranking clusters with respect to muscle mass, strength or function showed individuals with low grip strength had a higher proportion of cells in C0, suggesting a stressed/pre‐senescence/senescence state may contribute to impaired myoblast function and reduced muscle strength.

Subclustering at increased resolution and pathway enrichment analysis identified five C0 subclusters (C00–C04), of which C00 exhibited key pathways involved in OXPHOS and oxidative stress, suggesting differentiating or stressed myoblasts along with the presence of senescence marker genes (*CDKN1A* and *CDK2NA*), confirming C0 findings. C01 pathways linked to RNA splicing and transcriptional regulation suggest transcriptionally active progenitors possibly mediated through responses to cellular stress. C02 and C03 pathways were transcriptionally distinct. Pathways in C02 were linked to cytoskeletal reorganisation and cell migration, along with cytokine signalling, cell adhesion and ECM adhesion suggesting cells attempting/undergoing structural modifications or remodelling. C03 cells showed expression profiles of genes and pathways linked to cell cycle, suggesting components of cellular activation/proliferation. Interestingly, Cluster 04, a heterogeneous cluster with no defined pathways but with genes linked to ECM remodelling, RNA processing/translational activity and mild stress markers was mainly representative of only seven participants, which may represent a cellular repair/remodelling stress responsive/adaptive cell state which is participant specific. To further investigate proliferating cells in C0, it would be informative to look in differentiating cultures to ascertain the differentiation trajectories of these cells post proliferation, examining if these trajectories indicate potential to differentiate towards specific myofibre types/subtypes with an altered/stressed phenotype and if they can be linked back to proliferating myoblasts with the C0 phenotype.

C1 showed high expression of myogenic markers, including *MYOD* and *MYF5*, along with later markers of differentiation, suggesting an activated myogenic programme with cells progressing towards differentiation. A higher proportion of cells in C1 was associated with individuals with high grip strength and ALMi, implying having a greater percentage of cells progressing towards myogenic differentiation is associated with increased muscle resilience and function. Enrichment of the 13 differentially expressed genes in C1 associated with grip strength in cell cycle‐mitotic, positive regulation of cytoskeleton organisation and chromatin remodelling pathways suggests that these may be important processes modulated during myoblast proliferation linked to muscle strength and health.

In contrast, cells in C2, C4 and C6 expressed the highest levels of cell proliferation markers, suggesting that they are actively dividing in the earliest stages of myogenic commitment. C4 and C6 also expressed many genes involved in DNA repair, including high expression of *BRCA1/2*, *PARPs* and *RAD21*. *BRCA1/2* are tumour suppressor genes that, alongside *RAD21* and *PARPs*, play key roles in DNA repair [23]. BRCA1 is linked to cellular resistance to DNA damage, limiting ROS production and oxidative stress in muscle cells [24]. High levels of *BRCA1* may protect myoblasts from oxidative damage as they undergo differentiation, switching from a highly glycolytic state to predominantly OXPHOS.

ALMi and grip strength were associated with higher proportions of cells within C6, suggesting that decreased myogenic commitment and/or stalled myogenic differentiation may contribute to low muscle mass and strength. In comparison, gait speed showed more marked differences in the relationship between cell clusters compared to grip strength or ALMi, with cells in C1 associated with lowest gait speed and cells in C6 associated with highest gait speed. Differences in associations between clusters and measures of muscle mass, strength and function suggest that impairments of different aspects of myoblast function contribute to each of the three definitional components of sarcopenia.

Cells within C3 expressed many components of the ECM, as well as *WNT5A* and *TGFB1*. Increased WNT signalling during ageing alters muscle stem cell fate with MUSCs from aged mice converting from myogenic to fibrogenic lineage, with increased expression of *COL1a1*, *FN1* and decreased expression of myogenic marker *MyoD* [25]. Cells in C3 associated with individuals with low ALMi showed increased *COL1a1* and *FN1* expression with decreased *MYOD* expression, suggesting that this cluster may represent myoblasts with high propensity to undergo myogenic to fibrogenic conversion. C5 expressed components of the ECM as well as β‐catenin, reported to play a dual role in myoblast differentiation, enhancing MyoD binding to myogenic loci and controlling cell–cell interactions preventing precocious/excessive myoblast fusion [26, 27]. C5 also expressed high levels of RNA binding proteins (RBPs), and enzymes involved in RNA processing, with RNA processing the most enriched pathway in this cluster. The post‐transcriptional regulation of gene expression orchestrated by RBPs plays crucial roles in muscle development and regeneration [28] with RBPs differentially associated with target mRNAs in proliferating myoblasts or differentiated myotubes where they regulate distinct aspects of RNA splicing in a stage‐dependent manner [29]. Individuals with the highest ALMi and grip strength had more cells in C1 and C5. It will be important to establish why this transcriptionally distinct population of myoblasts in C5 are associated with improved muscle function, whether they represent a particular stage of myogenic ECM remodelling or fusion and how they contribute to improved muscle function and mass.

C7–12 contained far fewer cells. With the exception of C10, cells in these clusters originated from the majority of participants, suggesting that they represent minor populations of myoblasts with distinct transcriptional heterogeneity. Cells within C7 and C12 expressed a high level of ECM components, C8 inflammatory genes, C9 cell morphogenesis markers and C11 myogenic genes. In contrast, cells in C10 came predominately from four participants, of which 66% originated from one participant and expressed high levels of *BGN*, *CD248* and *KRT7* fibroblastic markers. This is consistent with cell behaviour from this participant in vitro, with poor proliferation and differentiation into myotubes upon stimulation, suggesting a high propensity to undergo fibrogenic conversion.

Differences were observed in gene expression within different clusters in relation to ALMi, grip strength and gait speed, demonstrating impaired muscle ageing not only influences the proportion of cells in each cluster but also gene expression within clusters. ALMi was associated with differential expression of three genes in C0, including *TOM1*, an adaptor protein needed for maturation of autophagosomes and their fusion with lysosomes [30]. Differentiation of myoblasts into mature myotubes is accompanied by remodelling of the mitochondrial network involving both mitochondrial clearance and biogenesis, whereby early in the differentiation process autophagy is upregulated, followed by subsequent removal of mitochondria via SQSTM1‐mediated mitophagy. Mitochondria are then repopulated via peroxisome proliferator activated receptor gamma and coactivator 1 alpha (PPARGC1A/PGC‐1α) mediated biogenesis. Downregulation of *TOM1* may inhibit or reduce the ability of myoblasts to remodel the mitochondrial network and impair their differentiation. Cells in this cluster also exhibited high levels of *SQSTM1* (*p62*) that targets specific cargoes for autophagy [31]. Expression of *p62* is widely used as an indicator of autophagic flux [32]. High levels of *p62* may indicate reduced autophagic flux, further impairing mitochondrial remodelling and elimination of dysfunctional or damaged mitochondria, driving the cells in this cluster to a pre‐ or senescence phenotype [33].

The highest number of differentially expressed genes with respect to ALMi were in C2, associated with proliferative cells, suggesting differences in the proliferative nature of myoblasts from individuals with high ALMi compared to low ALMi. Interestingly, differentially expressed genes included *DNAJB6*, which interacts with chaperone proteins and has been implicated in myofibrillar myopathy [34]; *PABPN1*, a poly A binding protein involved in muscle wasting [35]; and *REXO2*, a nucleotide‐degrading enzyme that regulates mitochondrial transcription [36]. For grip strength, there were 13 differentially expressed genes in C1 including AURKB, transmembrane nucleoporin (NDC1), MYB proto‐oncogene like 2 (MYBL2), transcription factor 19 (TCF19), bromodomain adjacent to zinc finger domain 2B (BAZ2B) and kinesin family member 2C (KIF2C). These all function to promote cell division but were downregulated in C1 cells in individuals with low grip strength. As cells in C1 were progressing towards myogenic commitment and exiting the cell cycle, the downregulation of such genes may push cells towards early differentiation, potentially impairing myoblast maturation.

Examining the relationship between different cell clusters identified five inferred lineages, with all cells proceeding down a common lineage from C2 into C6 and four before diverging into five distinct lineages. Cells in L2 initially expressed cell proliferation markers, followed by early markers of myogenic differentiation and then later markers of muscle differentiation consistent with the myogenic programme being activated. Likewise, L5 initially expressed cell proliferation markers, which were then downregulated as cells progressed along the lineage; there was then upregulation of myogenic markers such as *Myf5*, *MyoD*, *H19* and *ACTN2*, which then declined alongside an upregulation of splicing components. Cells in L2 and L5 were less likely to be from individuals with low ALMi or grip strength, suggesting that having a greater frequency of cells within these two lineages is associated with increased muscle resilience, consistent with earlier data showing cells progressing towards myogenic differentiation (C1) and cells within the transcriptionally novel C5 (present in the latter part of L5) associated with higher grip strength and ALMi.

Interestingly, in L1, L3 and L4, cells either initially expressed cell proliferation markers alongside early myogenic markers and/or showed a transient peak in expression of myogenic markers before these were downregulated as cells advanced along the trajectory, suggesting that cells in early stages of these lineages were also progressing towards myogenic differentiation. However, the myogenic differentiation programme appeared to stall, with cells instead proceeding down alternative trajectories, for instance, cells progressing towards either an oxidative stressed state in L1, or a fibrogenic phenotype in L3 and L4. There were higher proportions of cells in L3 and L4 from individuals with low grip strength and ALMi, suggesting intrinsic defects in myoblasts from individuals with low ALMi and grip strength that attenuate the myogenic programme due to increased oxidative damage or transformation to a more fibrogenic phenotype. Kimmel et al. have suggested from studies on ageing MuSCs that myogenic differentiation proceeds through an unstable intermediate state, rather than transitioning through states of monotonically increasing stability [37]. These findings support this premise, suggesting that the myogenic differentiation programme can stall, and cells revert and/or proceed along several alternative trajectories. Understanding factors that influence cell trajectories and their differing fates will be critical to development of effective interventions.

There are several strengths to this study. Firstly, we carried out scRNAseq on a large number of cells from a sizeable cohort (132 older adults) compared to previous studies [14, 38]. The large sample size overcomes inherent variability seen in human studies, permitting generalisation of results to the wider population. Furthermore, we provide one of the first large scRNAseq datasets in older adults and have shown distinct heterogeneity of cultured myoblasts with differences in respect to grip strength, ALMi and gait speed; cells from individuals with low ALMi or low grip strength demonstrated a stalled myogenic commitment and propensity to convert to a fibrogenic‐like lineage or a pre‐senescence state respectively. Among limitations to this study, cell isolation and culture may influence the transcriptome, and due to the nature of the Drop‐seq process and number of cultures being analysed, cell cultures could not be cultured for an identical duration or starting from the same initial cell number; however, all cells were treated equally and at the same early passage number, with studies showing cultured myoblasts retain many phenotypic characteristics of individuals from which they were isolated [39, 40] and being frequently used in disease models [10]. To date, scRNAseq has mainly been performed on SCs freshly isolated from tissues, so our study cannot characterise transcriptional heterogeneity of quiescent SCs, but rather document transcriptional differences after isolation and culture in activated cells. As a result, quiescence markers were not observed; instead, myoblasts expressed cell proliferation and/or myogenic markers including Mi67 (late activation/proliferating MuSCs), Myf5 (early primed for activation MuSCs) and MyoG (differentiating‐MuSCs), representing different stages of activation and commitment [15]. Furthermore, our study shows distinct differences between myoblasts isolated and cultured from individuals with low grip strength, ALMi and gait speed, providing valuable insights into how loss of muscle strength, mass and function may affect latter stages of myoblast commitment and function. Our study suggests that myoblasts can proceed down a number of transcriptional trajectories; however, whether such differences in transcription are reflected by changes in the proteome or myoblast function is currently unknown. Furthermore, factors that induce the transcriptional heterogeneity seen here are unknown and further studies are required to determine drivers of such changes. However, the demonstration that such transcriptional heterogeneity exists, and identification of pathways altered in myoblasts from individuals with low muscle strength, mass or function is the first step in understanding causality of such changes.

### 4.1 Conclusion

Our single‐cell analysis demonstrates considerable myoblast transcriptional heterogeneity, with myoblasts from individuals with low muscle mass and strength progressing towards fibrogenic or stressed states, respectively. We have provided increased resolution and understanding of transcriptional changes that occur in individuals with low later life muscle strength, mass and function, which is critical for the development of strategies to improve muscle health.

## Funding

This work was supported by grant funding from the Medical Research Council (MC_U47585827, MC_ST_U2055, MC_PC_21003 and MC_PC_21001), Arthritis Research UK, Royal Osteoporosis Society, International Osteoporosis Foundation, Cohen Trust, NIHR Southampton Biomedical Research Centre, University of Southampton and University Hospital Southampton NHS Foundation Trust, NIHR Musculoskeletal Biomedical Research Unit and the University of Oxford. K.M.G. is supported by the UK Medical Research Council (MC_UU_20/4), the US National Institute of Ageing of the National Institutes of Health (award number U24AG047867) and the National Institute for Health Research (as an NIHR Senior Investigator [NF‐SI‐055‐0042] and through the NIHR Southampton Biomedical Research Centre [NIHR203319]) and Alzheimer‘s Research UK (ARUK‐PG2022A‐008). K.A.L. was supported by Rosetrees Trust, Wessex Medical Trust and Rank Prize. H.P.P. is supported by the National Institute for Health Research through the NIHR Southampton Biomedical Research Centre. M.B. is supported by the Vivensa Foundation.

## Ethics Statement

The authors of this manuscript certify that they comply with the ethical guidelines for authorship and publishing in the *Journal of Cachexia, Sarcopenia and Muscle*. The study was approved by the Hertfordshire Research Ethics Committee (07/Q0204/68) and has therefore been performed in accordance with the ethical standards laid down in the 1964 Declaration of Helsinki and its later amendments. All participants gave their written informed consent prior to their inclusion in the study.

## Conflicts of Interest

K.M.G. and H.P.P. have received reimbursement for speaking at conferences sponsored by companies selling nutritional products. C.C. has received consultancy fees and honoraria from Amgen, Danone, Eli Lilly, GlaxoSmithKline, Medtronic, Merck, Nestlé, Novartis, Pfizer, Roche, Servier, Shire, Takeda and UCB. N.C.H. has received consultancy/lecture fees/honoraria/grant funding from Alliance for Better Bone Health, Amgen, MSD, Eli Lilly, Radius Health, Servier, Shire, UCB, Consilient Healthcare, Kyowa Kirin, Theramex and Internis Pharma. M.A.B., E.S.G., E.A., K.M.G. and K.A.L. are part of academic research programmes that have received research funding from BenevolentAI Bio Ltd., Nestec and Danone. The other authors declare no conflicts of interest.

## Supporting information


**Data S1:** Supporting Information.

**Figure S1:** Violin plots of Cluster 8, which contained 400 cells and expressed high levels of inflammatory genes, showing interleukin‐6 (*Il‐6*), interleukin‐8 (*Il‐8*) and nuclear factor kappa light chain enhancer of activated B cells (*NFkB2*).
**Figure S2:** Dot plots and associated violin plots of *BGN*, *KRT7* and *CD248* in Cluster 10.
**Figure S3:** Proportion of myoblasts in each cell lineage (1–5) with respect to the four quartiles of ALMi (A), grip strength (B) and gait speed (C).

**Table S1:** Descriptive statistics at HCS baseline (1998–2004) for the HSSe analysis sample and for HCS participants who were not included in the HSSe analysis sample (men and women pooled).
**Table S2:** Analysis of batch effect in single cell data.
**Table S3:** Cell counts per cluster.
**Table S4a:** Marker genes for each cell cluster.
**Table S4b:** Marker genes for Cluster 0 subclusters 00–04.
**Table S4c:** Pathway analysis for Cluster 0 subclusters 00.
**Table S4d:** Pathway analysis for Cluster 0 subclusters 01.
**Table S4e:** Pathway analysis for Cluster 0 subclusters 02.
**Table S4f:** Pathway analysis for Cluster 0 subclusters 03.
**Table S5:** Top 3 enriched GO biological process (BP) and molecular function (MF) pathways for marker genes in Clusters 0–6.
**Table S6:** Associations between ALMi, grip strength and gait speed and cell clusters.
**Table S7a:** Differential gene expression within clusters with respect to ALMi, grip strength and gait speed.
**Table S7b:** Pathway analysis (Metascape) of the 13 differentially expressed genes associated with grip strength.
**Table S8:** Differential gene expression at start and end of each lineage.
**Table S9:** Differences in cell proportions within each lineage with regard to four quartiles of ALMi, grip strength and gait speed.
