# Supplementary material for: Single‐Cell RNAseq Identifies Heterogeneity in Myoblasts From Older Adults With Differences Related to Muscle Mass and Function
Source: J Cachexia Sarcopenia Muscle. 2026 Feb 17;17(1):e70213. doi: 10.1002/jcsm.70213 (PMC12913704; doi:10.1002/jcsm.70213)
Supplement: Supplementary file 1 — Data S1: Supporting Information. [file JCSM-17-e70213-s002.docx]

**Supplementary Methods**

**Isolation of Satellite cells from Muscle Biopsies and Myoblast Culture**

Percutaneous muscle biopsies of the vastus lateralis were conducted after an overnight fast under local anaesthetic using a Weil-Blakesley conchotome and placed in room temperature PBS. Biopsies were minced, digested in 0.5 mg/ml collagenase (Sigma) at 37°C for 20 min, before being filtered through a 100µM cell strainer (BD Falcon). Cells were pre-plated to remove fibroblasts, before sorting using CD56 MicroBeads (Miltenyi Biotech) prior to experiments ^1^. Cells were grown in proliferation medium (DMEM containing 20% fetal bovine serum (FBS), 10% Horse Serum (HS), 1% Chick embryo extract (CEE) (Sera Labs) and 1% Penicillin/Streptomycin (P/S)). Cells were collected at passage 4 for scRNAseq.

**Cell Collection, Encapsulation and Reverse Transcription (Single-cell Drop-seq pipeline)**

Myoblast cultures were thawed in three separate culture batches for experiments. Cells were grown to approximately 60% confluence before collection and trypsinised (0.25% trypsin-EDTA). Trypsin was quenched with complete proliferation media and cells counted (Fuchs-Rosenthal haemocytometer). Cells were centrifuged (*1000g*) for 5 minutes and resuspended in PBS+0.01% BSA at 100 cells/µl. Barcoded Macosko beads ^2^ (ChemGenes) were resuspended in lysis buffer (200mM Tris pH 7.5, 6% Ficoll PM-400, 0.2% Sarkosyl, 20mM EDTA, 50mM DTT) at a concentration of 100 beads/µl. Bead suspension was loaded into a 3ml plastic syringe, cell suspension into a 1ml plastic syringe and droplet generation oil (Biorad) loaded into a 5ml syringe. Beads were kept in suspension by a magnetic stirrer. Solutions were injected into a microfluidic device (Thistle Scientific) at a flow rate of 15,000µl/hr (oil) and 4,000µl/hr (beads and cells). Droplets were examined under a microscope to ensure uniformity and single bead occupancy prior to collection. After collection, excess oil was removed, 6X SSC buffer added along with 1ml Perfluorooctanol (PFO) (Sigma). Droplets were broken by vigorous shaking (by hand) for 20 seconds. Samples were centrifuged (*1000xg*) for 1 minute and supernatant removed. 30ml 6X SSC was added and the aqueous layer transferred to a new tube. Samples were centrifuged (*1000xg*) for 1minute and bead pellet transferred to an ultra-low binding1.5ml Eppendorf. Beads were washed with 6X SSC and with 5X Maxima H- RT Buffer prior to reverse transcription in which200µl RT mix, consisting of 1X Maxima RT buffer, 4% Ficoll PM-400, 1mM dNTPs (Clontech), 1U/µl RNase Inhibitor (Lucigen), 2.5µM Template Switch Oligo and 10U/µl Maxima H- RT, was added to the beads and incubated with rotation at room temperature for 30 minutes, followed by 90 minutes at 42°C.

**Exonuclease I Treatment and cDNA Amplification (Single-cell Drop-seq pipeline)**

Beads were washed once with Tris-EDTA-Sodium Dodecyl Sulfate (TE-SDS), twice with Tris-EDTA-Tween (TE-TW) and once with 10mM Tris pH7.5, resuspended in 200µl Exonuclease I mix consisting of 1X Exonuclease I buffer and 1U/µl Exonuclease I (NEB) and incubated for 45 minutes at 37°C with rotation. Beads were washed once with TE-SDS, twice with TE-TW, once with H_2_O and counted (Fuchs-Rosenthal haemocytometer). For each sample, 5x PCR reactions, each with 2000 beads, were amplified in a 50µl mix consisting of 1X KAPA HiFi HotStart Readymix (Kapa Biosystems) and 0.8µM SMART PCR Primer. Thermocycling conditions were as follows: 95°C for 3 minutes; 4 cycles of: 98°C for 20 seconds, 65°C for 45 seconds and 72°C for 3 minutes; 9 cycles of: 98°C for 20 seconds, 67°C for 20 seconds and 72°C for 3 minutes; finally followed by 72°C for 5 minutes. Libraries were purified with 0.6X AMPure XP beads (Beckman Coulter) following the manufacturer’s protocol and eluted in 13µl of H_2_O. The concentration was quantified on a BioAnalyzer High Sensitivity DNA Chip (Agilent) and the 5x PCR reactions for each sample were pooled at equimolar amounts.

**Transposase Fragmentation and Selective PCR (Single-cell Drop-seq pipeline)**

For each sample, 500pg purified pooled cDNA was used as input to the Nextera XT tagmentation reaction (Illumina). Tagmentation was carried out following the manufacturer’s protocol, except for 200nM custom primer P5 SMART PCR Hybrid oligo and a custom barcoded i7 primer. Samples were amplified as follows: 95°C for 30 secs; then 12 cycles of: 95°C for 10 seconds, 55°C for 30 seconds, 72°C for 30 seconds; followed by a final 5 minutes at 72°C. Tagmented libraries were purified with 0.6X AMPure XP beads following the manufacturer’s protocol and eluted in 10µl H_2_O. Library concentration was quantified on a BioAnalyzer High Sensitivity DNA Chip. All libraries were pooled to a final concentration of 250pM and were sequenced by Oxford Genomics Centre (OGC) (https://www.well.ox.ac.uk/ogc/) on the Illumina NovaSeq 6000 using 0.3µM of Custom Read 1 primer. Read 1 was 20bp and read 2 was 75bp.

**Pre-processing of Single-cell RNA-seq data and DGE matrix filtering**

FASTQ files were passed through the Dropseq alignment pipeline^2^. Briefly, FASTQ files were converted into unmapped BAM files using Picard’s FastqToSam function and sorted by read name using SortSam (Broad Institute). Reads were tagged with the cell and molecular barcodes, adding an XM tag for molecular barcodes and XC tags for cell barcodes. Reads with low quality bases in the cell or molecular barcodes were removed and any SMART adapter sequence or trailing polyA tails were trimmed. BAM files with cell and molecular barcodes extracted were converted back to FASTQ files and aligned to the hg19 genome using the STAR aligner. Aligned BAM files were sorted by read name and the aligned and unaligned BAM files were merged using Picard’s MergeBamAlignment tool (Broad Institute), to combine alignments with their cell and molecular barcodes. Reads were tagged with the BAM tag GE for reads which overlap the exon of a gene as provided by the hg19 refFlat file. Finally, a digital gene expression (DGE) profile was generated for each sample, with the gene expression of each cell per sample.

**Cell cycle scoring**

Cell cycle scores were generated using the CellCycleScoring function from the Seurat package v3.2.2 ^3^. Briefly, cell-cycle scores were generated based on the expression levels of previously published G2M and S phase gene signatures. These marker sets should be anticorrelated in their expression levels, and cells expressing neither are most likely in G1 phase. G2M and S phases were assigned based on the highest positive score, while G1-phase was assigned if both G2M and S-phase scores were < 0. As some cells may have progressed to myogenic differentiation while others were proliferating, we adjusted the data for the difference between the G2M and S phase scores, such that the signals separating non-cycling and cycling cells was maintained, but differences in cell cycle phase among proliferating cells was be adjusted for.

**Dimensionality reduction**

Dimensionality reduction was carried out using Seurat v3.2.2^3^. Log-normalized counts for each cell (*E_i,j_*) were calculated by dividing counts for gene *i* by the total counts in cell *j*, multiplied by 10,000, after which ln(*E_i,j_*+1) was calculated. Number of features per cell (nFeature_RNA), cell cycle score difference, proportion of mitochondrial UMIs and participant sex were adjusted by regressing each variable against each feature, and the resulting residuals were scaled and centred. The 2000 most variable genes were determined using the variance stabilising transformation method and used in downstream analyses. RunPCA was used to compute principal components of which the top 50, as identified using a scree plot, were used downstream.

Ambient RNA was estimated using DecontX ^4^. DecontX is a Bayesian method which assumes the observed expression of a cell is a mixture of counts from two multinomial distributions; a distribution of native counts from the cell’s actual population and a distribution of contaminating transcripts from all other cell populations captured in the assay. DecontX performs heuristic clustering to quickly define major cell clusters, and genes that are more highly expressed in other cell populations will be more likely to contribute to contamination in the current cell population. Therefore, these genes will have relatively higher probabilities in the contamination distribution compared to the expression distribution in native cell population and counts for these genes will be more likely to be called “contamination.” After identification of contaminating counts for each cell, the contaminating background RNA identified for each cell by DecontX was summed to obtain the contamination at the sample level. This background RNA count vector was transformed for each sample as if it were the count vector for a cell and projected into the PCA space computed from the real cells. The dot product of each real cell PCA coordinate vector with each sample’s background vector was computed, generating the ‘background loading’ of a given cell for a given sample ^5^. Finally, a linear regression model was fitted – real cell PCA coordinate matrix$\sim$cell background loadings – and the residuals of the models is the background corrected PCA matrix. The UMAP algorithm was used to reduce the dimensionality of the background corrected PCA matrix and used for cell clustering and data visualisation.

**Single-cell RNA-seq data clustering**

Clustering was performed using Seurat v3.2.2. Briefly, FindNeighbors function was used to compute k nearest neighbours. The clustree package was used to determine the resolution between 0.2 and 1 with Louvain clustering. A final resolution of 0.5 was used to cluster the cells. Differential expression and marker genes between clusters were identified by Wilcoxon rank sum test.

**Pathway analysis**

Pathway analysis of the 13 differentially expressed genes associated with grip strength and subclusters of C0 were performed using Metascape, with the minimum gene overlap set at 3, minimum enrichment value set at 1.5 and a p value cutoff of 0.05 ^6^.

For bioinformatics analysis methodologies, aspects of bespoke code can be made available on request to the corresponding author. Sequencing data can be made available through contacting the corresponding author.

**References**

1. Agley, C.C., Rowlerson, A.M., Velloso, C.P., Lazarus, N.L. & Harridge, S.D. Isolation and quantitative immunocytochemical characterization of primary myogenic cells and fibroblasts from human skeletal muscle. *J Vis Exp*, 52049 (2015).

2. Macosko, E.Z.*, et al.* Highly Parallel Genome-wide Expression Profiling of Individual Cells Using Nanoliter Droplets. *Cell* **161**, 1202-1214 (2015).

3. Stuart, T.*, et al.* Comprehensive Integration of Single-Cell Data. *Cell* **177**, 1888-1902 e1821 (2019).

4. Yang, S.*, et al.* Decontamination of ambient RNA in single-cell RNA-seq with DecontX. *Genome Biol* **21**, 57 (2020).

5. Packer, J.S.*, et al.* A lineage-resolved molecular atlas of C. elegans embryogenesis at single-cell resolution. *Science* **365**(2019).

6. Zhou, Y.*, et al.* Metascape provides a biologist-oriented resource for the analysis of systems-level datasets. *Nat Commun* **10**, 1523 (2019).
