# Supplementary material for: Single‐Cell RNAseq Identifies Heterogeneity in Myoblasts From Older Adults With Differences Related to Muscle Mass and Function
Source: J Cachexia Sarcopenia Muscle. 2026 Feb 17;17(1):e70213. doi: 10.1002/jcsm.70213 (PMC12913704; doi:10.1002/jcsm.70213)
Supplement: Supplementary file 2 — Figure S1: Violin plots of Cluster 8, which contained 400 cells and expressed high levels of inflammatory genes, showing interleukin‐6 (Il‐6), interleukin‐8 (Il‐8) and nuclear factor kappa light chain enhancer of activated B cells (NFkB2). Figure S2: Dot plots and associated violin plots of BGN, KRT7 and CD248 in Cluster 10. Figure S3: Proportion of myoblasts in each cell lineage (1–5) with respect to the four quartiles of ALMi (A), grip strength (B) and gait speed (C). [file JCSM-17-e70213-s003.docx]

Supplementary Figures


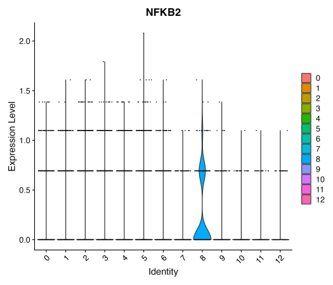

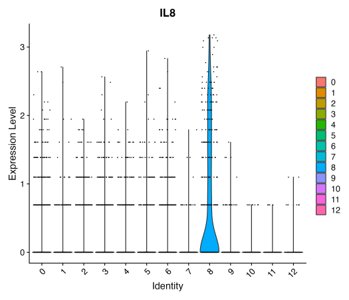

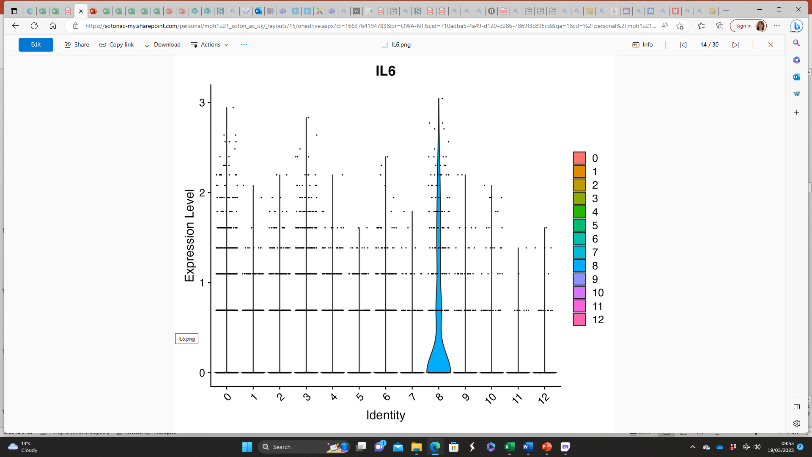


**Supplementary Figure 1**: Violin plots of Cluster 8 which contained 400 cells and expressed high levels of inflammatory genes, showing Interleukin-6 (*Il-6*), Interleukin-8 (*Il-8*) and **Nuclear Factor Kappa-Light-Chain-Enhancer of activated B cells** (*NFkB2*).


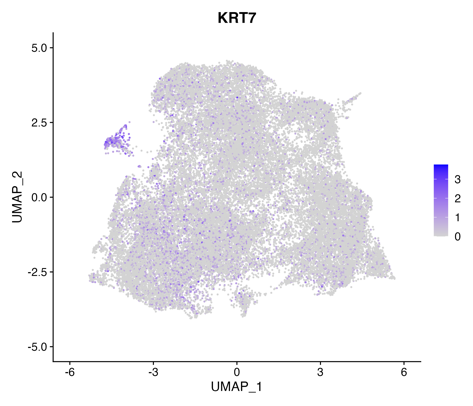

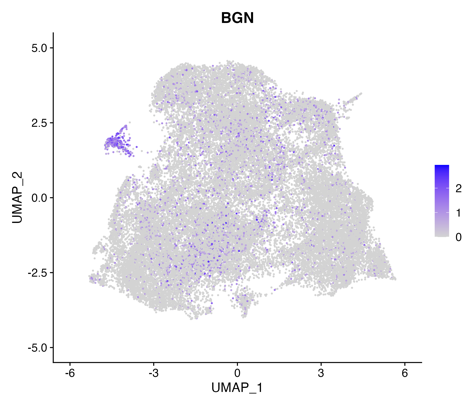

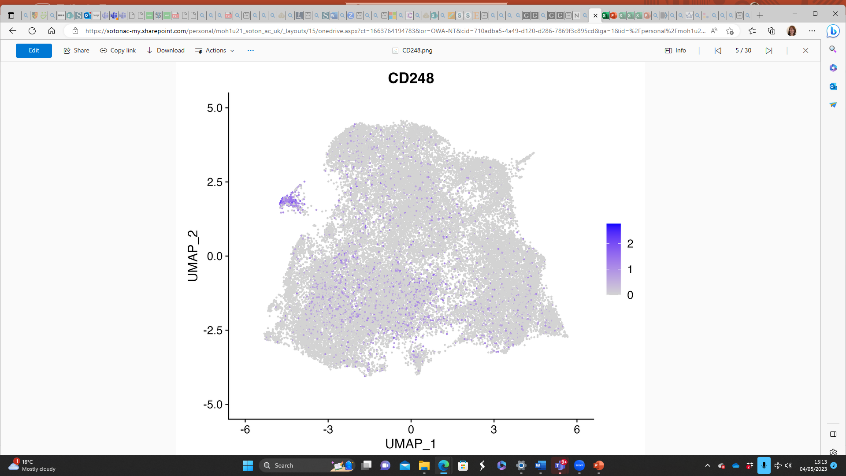


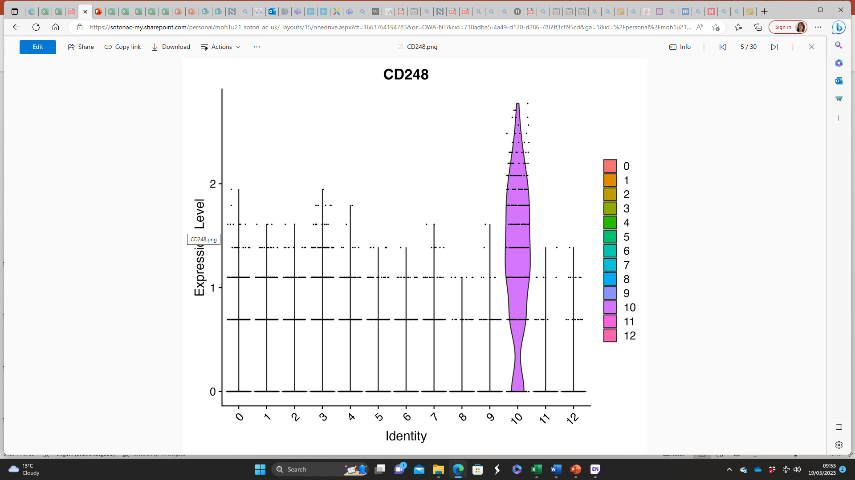

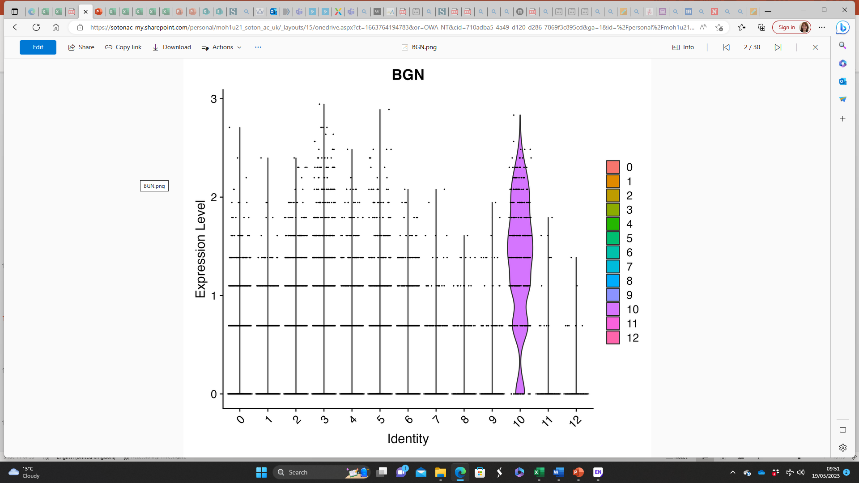

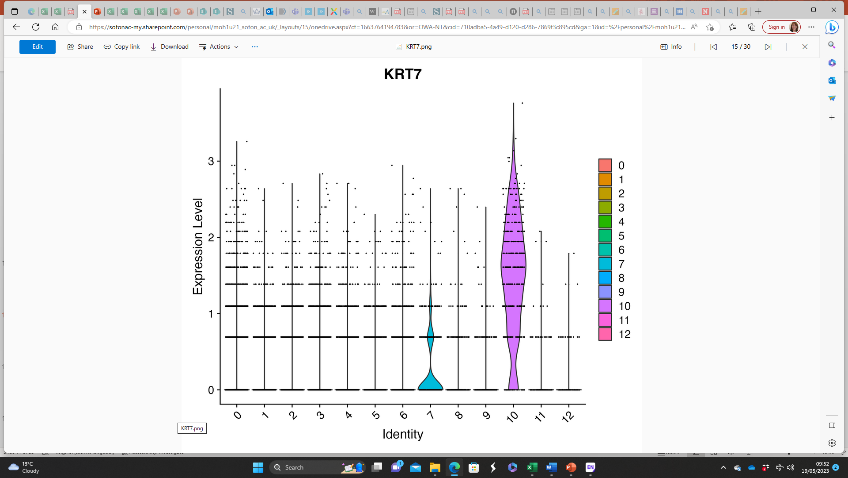


**Supplementary Figure 2**: Dot plots and associated Violin plots of *BGN*, *KRT7* and *CD248* in Cluster 10.

**
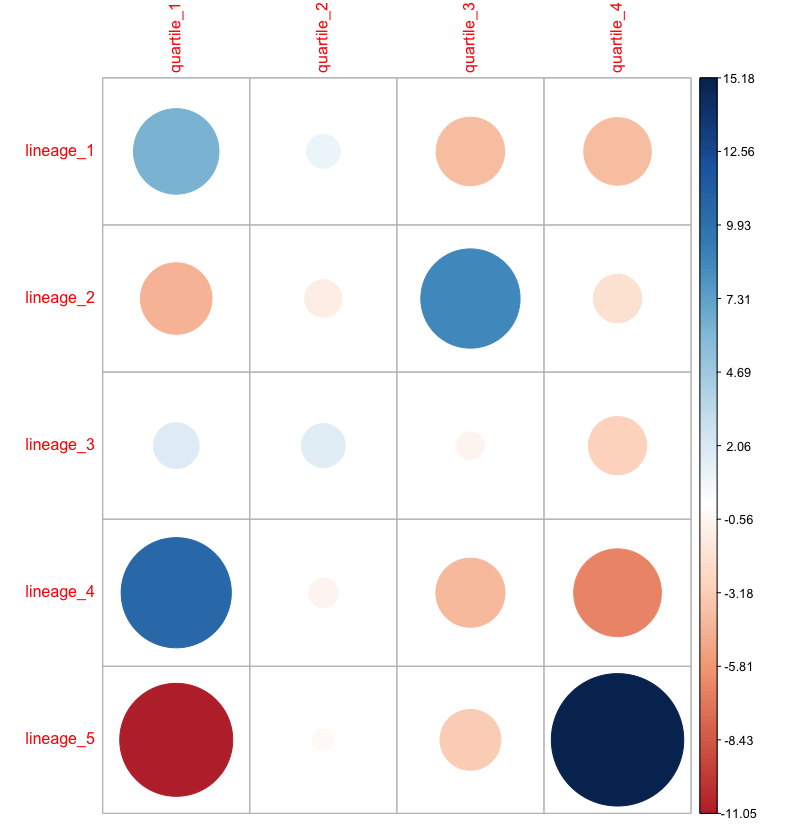

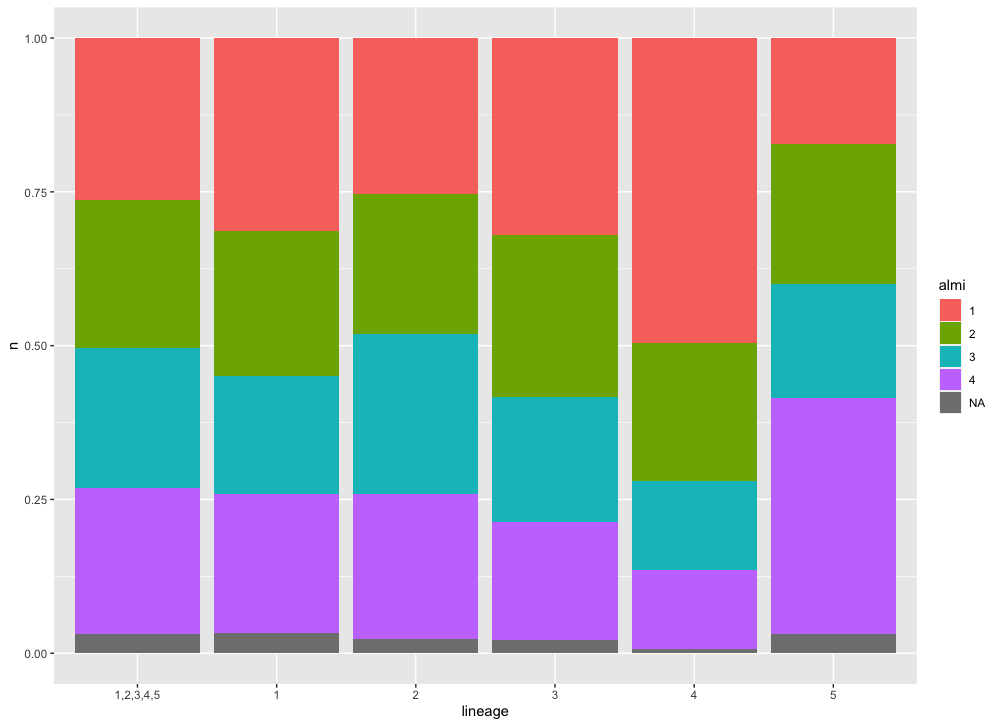
A**

**
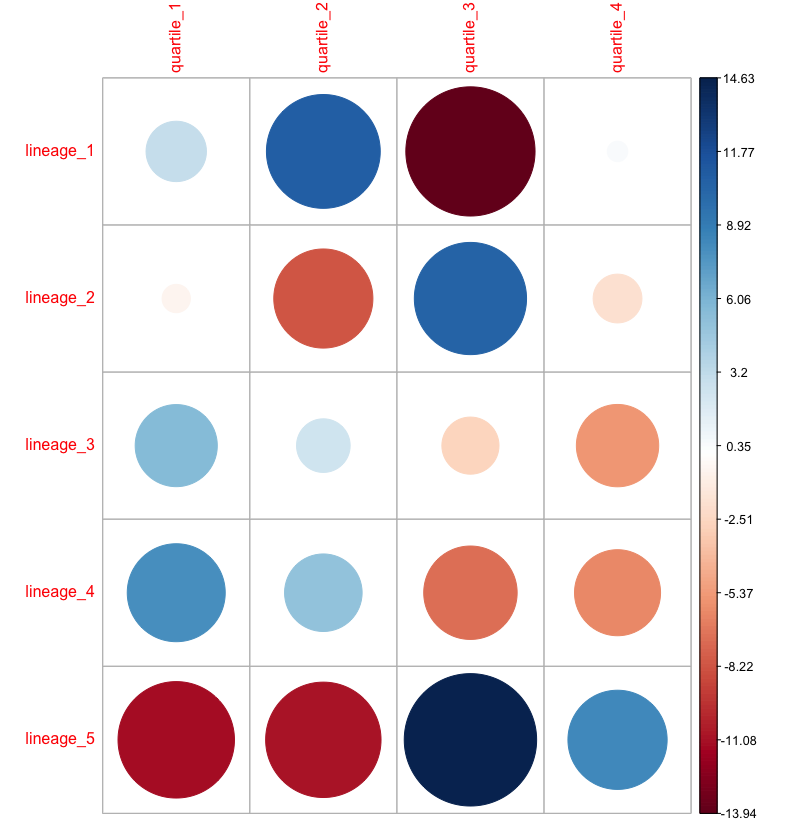
**

**
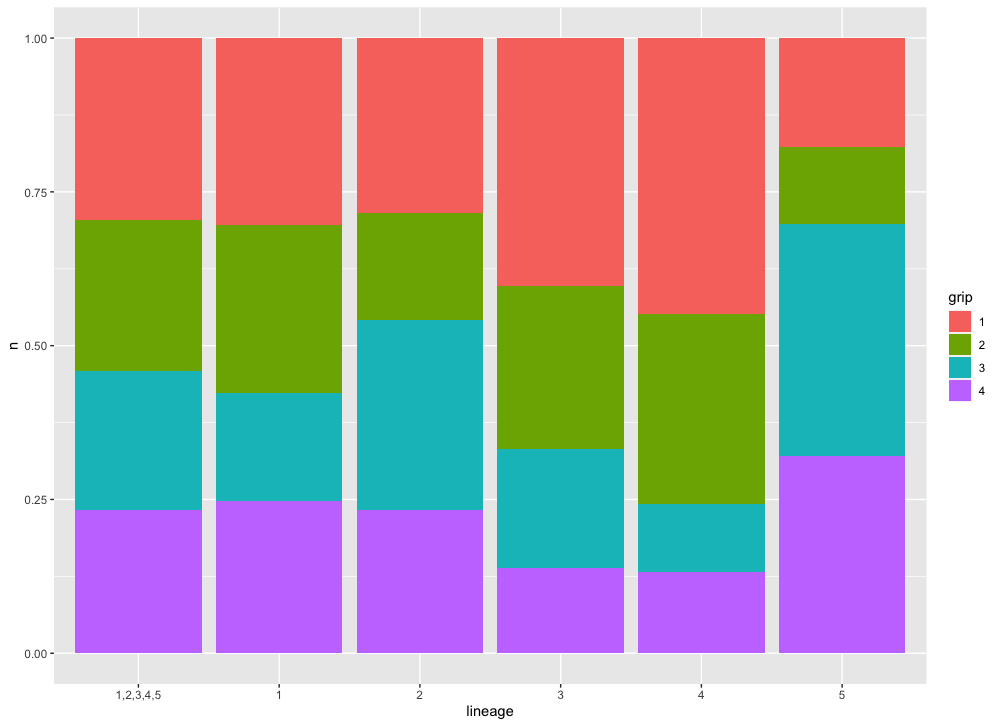
B**

**
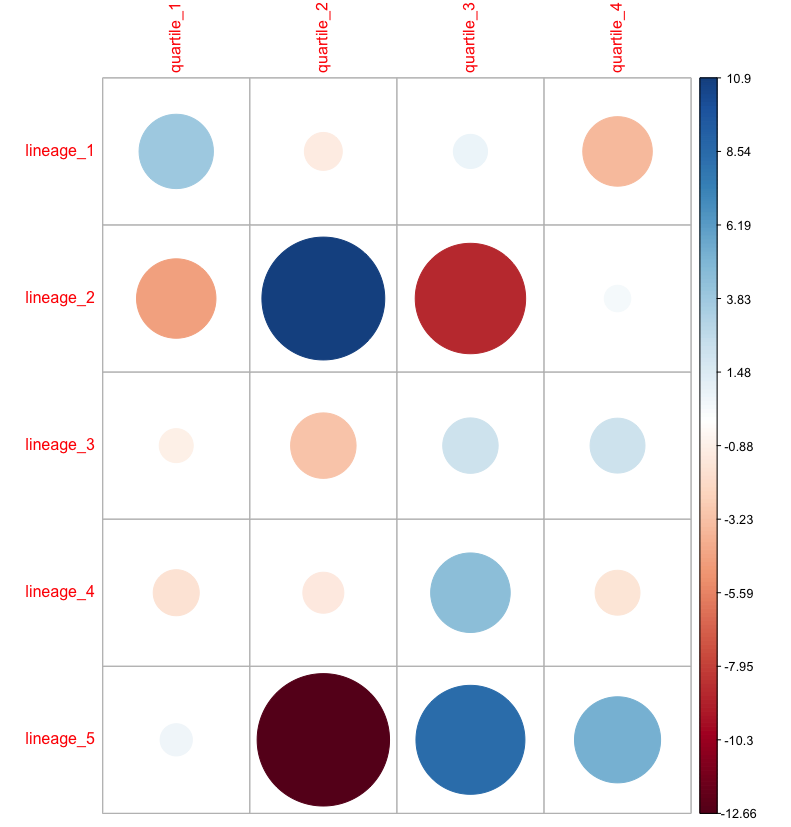
**
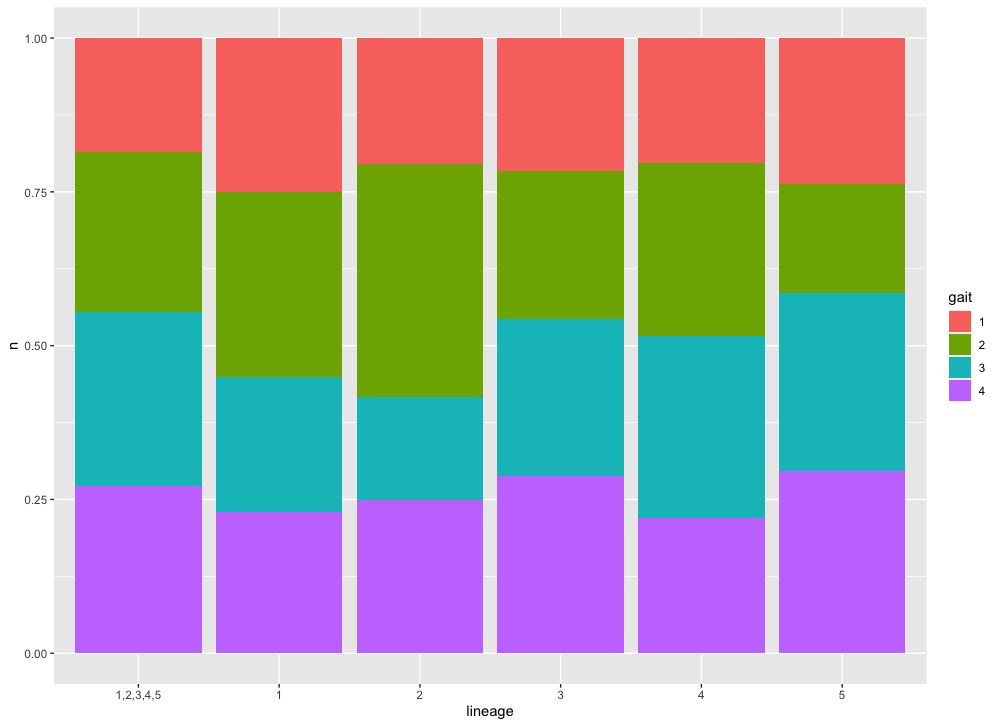
**C**

**Supplementary Figure 3:** Proportion of myoblasts in each cell lineage (1-5) with respect to the 4 quartiles of ALMi (**A**), Grip Strength (**B**) and Gait speed (**C**).
